# Estimating carbon footprints from large scale financial transaction data

**DOI:** 10.1111/jiec.13351

**Published:** 2022-12-27

**Authors:** Anna Trendl, Anne Owen, Lara Vomfell, Lena Kilian, John Gathergood, Neil Stewart, David Leake

**Affiliations:** 1 Behavioural Science, Lloyds Banking Group, 25 Gresham Street, London, EC2V 7HN UK; 2 Warwick Business School, University of Warwick, Coventry, UK; 3 School of Earth and Environment, University of Leeds, Leeds, UK; 4 Leeds Institute for Data Analytics, University of Leeds, Leeds, UK; 5 Centre for Spatial Analysis and Policy, School of Geography, University of Leeds, Leeds, UK; 6 School of Economics, University of Nottingham, Nottingham, UK

**Keywords:** big data, carbon footprint, consumption, greenhouse gas emissions, household expenditure, industrial ecology

## Abstract

Financial transactions are increasingly used by consumer apps and financial service providers to estimate consumption‐based carbon emissions. This approach promises a low‐resource, ultra‐fast, and highly scalable approach to measuring emissions at different levels of potential policy intervention—spanning the national, subnational, local, and individual level. Despite this potential, there is a lack of research exploring the validity of this approach to carbon profiling. Here we address this oversight in three ways. First, we provide a step‐by‐step description of our approach toward estimating carbon footprints from micro‐level transaction data generated by more than 100,000 customers of a large retail bank in the United Kingdom. Second, we quantitatively compare emission estimates obtained from transaction data with those calculated from a more standard data source used in carbon profiling, the largest household expenditure survey in the United Kingdom. Third, we offer a detailed qualitative comparison of the advantages and disadvantages of transactions versus alternative data sources (such as survey data), across key dimensions including data availability, data quality, and data detail. We find that financial transactions offer a credible alternative to survey‐based sources and, if made more widely accessible, could provide important advantages for profiling emissions. These include objective, micro‐level data on consumption behaviors, larger sample sizes, and longitudinal, frequent data capture.

**Supplementary Information:**

The online version of this article (doi:10.1111/jiec.13351) contains supplementary material, which is available to authorized users.

## INTRODUCTION

In recent decades, accounting for greenhouse gas (GHG) emissions from the perspective of consumption has moved up the economic and political agenda. Working Group III's contribution to the Intergovernmental Panel on Climate Change's fifth assessment report has a chapter exploring consumption growth as a driver for GHG emissions (Blanco et al., 2015). In the United Kingdom, the Climate Change Committee (CCC) specifically recommended that UK consumption emissions be “monitored….closely” (Climate Change Committee, 2019, p. 162). Measuring consumption‐based emissions at different levels of potential policy intervention—national, subnational, local, and even at the individual level—is a critical requirement for the successful implementation of climate policies to deliver on Net Zero.

Despite this pressing need, clear consensus is yet to emerge on appropriate data sources and calculation methodologies for disaggregated, consumption‐based emissions. We see three broad approaches currently in circulation. The first involves combining household spend data from national surveys with carbon emission factors derived from environmentally extended multiregional input–output (MRIO) databases (Tukker et al., 2018; Wiedmann et al., 2011). Household expenditure surveys, such as the Living Cost and Food Survey (LCFS) in the United Kingdom (Office for National Statistics, 2021), provide an annual view of consumption at the household level, and have been used to study how emissions vary by key demographic characteristics (Owen & Barrett, 2020). Whilst household surveys represent an important source of insights for climate policy, there are significant limitations, such as the low frequency of data collection and relatively modest sample sizes.

A second approach, also involving data collection via surveys, focuses instead on specific activities that generate known physical quantities of emissions (Carbon Footprint Calculator, 2022; Streamlined Energy and Carbon Reporting in the UK, UK Government, 2019; WWF UK, 2022). This approach can be highly accurate as it relies on measuring actual activity, rather than proxying activities with more general sources of information, such as data on household spending. It also provides emission measurements at the individual level. However, completing detailed surveys is time consuming and requires participants to track down a broad range of information that may not be readily available. These frictions and the associated costs to incentivize can result in low participation rates. Biased samples may also be a concern, due to participants self‐selecting to take part (West et al., 2016).

A third approach, which we see becoming increasingly popular, involves estimating emissions based on peoples’ spending activity as recorded via financial transactions captured by payments technology. As we describe in detail in this paper, by combining information from financial transactions with MRIO‐based carbon emission factors, detailed carbon emission profiles can be constructed. A range of consumer apps are emerging using this core approach (see Svalna, 2022; CoGo, 2022; Earthchain, 2022; ecolytiq, 2022), promising low‐resource, ultra‐fast, and highly scalable emission measurements. Despite the potential opportunity and growing prevalence of the approach, to our knowledge, there are as‐of‐yet no formal research studies exploring the validity of transaction‐based emission estimates and its advantages and disadvantages over alternative approaches.

Here, we begin to address this gap in the research literature in three ways. First, we provide a detailed overview of the steps involved in calculating carbon footprints from micro‐level transaction data generated by more than 100,000 customers of a large retail bank in the United Kingdom. Second, we quantitatively compare carbon emission estimates produced by financial transaction data to those calculated from a household spend survey, demonstrating that transaction‐based emission estimates correspond closely to those obtained from the largest household spend survey in the United Kingdom (LCFS). We provide these comparisons across several demographic variables and consumption categories.

Third, we offer a detailed qualitative comparison of the advantages and disadvantages of using transactions versus alternative sources, across dimensions including data availability, data quality, and data detail. This review finds that transaction data, if made more widely accessible, can offer important advantages over other methods, including objective, micro‐level data on consumption behaviors; larger sample sizes; and longitudinal, more frequent data capture. In contrast, surveys often lack these characteristics and self‐reported data may be biased by individual attention and preferences.

Based on these analyses, we conclude that transaction data offers a valuable additional data source for climate policy makers. Transaction data also offers individuals, businesses, and other organizations easier access to their own carbon footprints, without the frictions imposed by complex survey questionnaires or expensive engagements with third‐party providers. This can encourage more informed consumer choices and support an evidence‐based transition to Net Zero.

The paper proceeds as follows. Section 2 describes our data sources and methodology for measuring emissions from transactions. Section 3 quantitatively compares our results to emission estimates obtained from the LCFS. Section 4 provides a qualitative evaluation of the benefits and challenges involved in using transactions compared to other data sources, while Section 5 discusses options available to improve data access.

## DATA AND METHODS

To estimate carbon footprints from financial transactions, we collect 2018 spend data from an anonymous sample of 101,744 customers from one of the United Kingdom's largest retail banks (Lloyds Banking Group, 2022; LBG). A transaction is defined as any spend that occurs on a personal current or credit card account, including electronic transfers (automated or not), online transactions, in‐store transactions, and ATM withdrawals. Full details on our sample selection procedure are provided in Section 2.2.1.

Our objective is to compare emissions calculated from these transactions to those from the LCFS, a long‐running survey of the weekly expenditures of UK households (Office for National Statistics, 2021), that has been previously used for measuring UK emissions (Owen & Barrett, 2020).

### LCFS data

Like other household spend surveys around the world, the LCFS is used to formulate retail price indices, analyze the effects of taxation and benefits, and to address other policy‐relevant topics such as trends in nutrition. As described elsewhere in the literature (Bulman et al., 2017), approximately 5500 households take part in the survey each year, with survey results weighted to be representative of the UK population.

While the LCFS collects household‐level spend data, it is possible to use these data to calculate individual‐level estimates using a process known as equivalization (Gough et al., 2011). We apply a modified OECD equivalence scale, as preferred by UK government, to convert each survey entry into the equivalent spend if the household was a single adult^1^ from the 2018 LCFS (*N* = 5473). We also obtain data on each household's income group (1–10), UK NUTS 2 region, and the age of the household reference person.

### LBG data

#### LBG sample selection

While the LCFS obtains representative samples, LBG's customer base is not necessarily representative of the entire UK population, though LBG is one of the UK's largest retail banks. In particular, it is estimated that 22% of UK adults hold current accounts across multiple financial service providers (Moon et al., 2015). Therefore, to ensure a fair comparison to the LCFS profiles which capture complete household expenditures by design, we set three criteria for LBG sample inclusion with the aim to restrict the sample to individuals who undertake their main banking activity with LBG.

First, we identify customers who processed at least 12 customer‐initiated transactions (e.g., non‐automated payments) in every month of 2018 (across current accounts and credit cards, if applicable). This first criterion filters out customers who may not use LBG as their primary provider or have recently switched providers. This filtering is standard practice within the bank.

Second, we restrict our sample to those who have at least one payment classified as “Energy” by LBG's internal Transaction Classification System (TCS)^2^. This is another indicator of a customer's primary bank account status and ensures our sample is directly comparable with the LCFS, which explicitly collects data on household energy spending. In addition, domestic energy spend is the largest component of a household's carbon footprint and needs to be accounted for to give an accurate measure of emissions (Minx et al., 2013).

This second criteria may exclude renters whose rent payments are bundled with household bills, and therefore for whom we are unable to estimate energy‐based emissions. However, this type of bundled bill structure is relatively rare in the United Kingdom (Property Reporter, 2022), and overall energy spend is unlikely to differ systematically by tenancy agreement (bills included/rent only) or tenure (renters/homeowners).

Third, we exclude individuals who have transfers to non‐LBG current accounts in their name, or payments toward non‐LBG credit cards exceeding 2.5% of their overall spending. Again, these criteria identify non‐primary account status. The 2.5% threshold is selected to avoid unnecessarily limiting our sample but to ensure we capture the majority of an individuals’ expenditure.

From this final sample of qualifying individuals, we draw an anonymous random sample of 101,744 aged 18 or over and identify their transactions associated with all open credit card and personal current accounts in 2018. Overall, 50% of individuals in this sample are found to hold a joint account (*N* = 50,744). The expenditure of individuals with joint accounts is likely to reflect household‐level spending, and we take this distinction into account in subsequent analyses (see Section 3).

#### LBG spend data

Of the 62 million transactions retrieved, the majority (86%) are categorized by the LBG TCS system or classified as ATM withdrawals. Of the 14% of transactions that are not classified, 51% are payments related to interest, charges, and fees, while 25% are inter‐account transfers (transfers between two LBG accounts).

We exclude transfers and payments that are not classified by TCS (except domestic ATM withdrawals), and the following TCS categories: unclassified transactions, overseas cash withdrawal, and payments toward other current accounts, credit cards, and savings accounts. The bank's TCS is structured hierarchically across three levels, with increasing granularity. For example, the highest, level 1, category of “Motor” spend has level 2 sub‐categories corresponding to “Petrol,” “Maintenance/Repair,” and “Parking/Tolls.”

Finally, we aggregated spend across all three levels of remaining TCS categories for these 101,744 individuals. The final list of TCS categories can be found in Supporting Information S5 (Table SI1).

### Carbon multipliers by COICOP category

To calculate carbon emission estimates from transaction data, we use “carbon multipliers.” These represent the emissions associated with £1 unit of spending in a given TCS category. We now describe their derivation in detail.

Carbon multipliers are constructed from two data sources. The first includes consumption‐based GHG emission estimates for the UK economy, disaggregated into sub‐categories (Department for Environment, Food & Rural Affairs, 2022). These originate from a MRIO framework. Here the UKMRIO is used, which reports carbon dioxide equivalents of GHGs.

Environmentally extended MRIO analyses enable indirect consumption‐based emissions of UK households to be calculated at a product level (Miller & Blair, 2009; Owen et al., 2018; Wood et al., 2019). Where relevant, direct household emissions from fuel burning are also included to construct more complete, total consumption‐based emissions.

The second data source contains total household‐level spending on different goods, provided by the LCFS. Crucially, both these data sources share a common categorization scheme (referred to as COICOP, the Classification of Individual Consumption by Purpose, United Nations, 2014). Therefore, by dividing emission estimates for a given COICOP category by its corresponding total household spending (kg CO_2_e/£), we can derive emissions associated with a £1 unit of spending.

These carbon multipliers by design account for both direct emissions associated with burning fuel to heat homes and driving cars, and indirect emissions, which consider the full domestic and international production supply chains of the goods and services consumed by UK households. A more detailed description of these two data sources can be found in the Supporting Information (SI).

Disaggregating national consumption‐based accounts using spend data, as done here, can introduce uncertainty where the same products vary in price. To reduce this uncertainty, some researchers rely on physical data to estimate emissions (Girod & de Haan, 2010; Goldstein et al., 2017; Hendrie et al., 2014; Jones & Kammen, 2014). However, detailed data on functional unit consumption are unavailable in many countries, including the United Kingdom. Therefore, even research that employs some physical measurements of consumption often relies on expenditure data (Vita et al., 2019).

Whilst a spend‐based approach may underestimate low footprints and overestimate high footprints, overall trends remain measurable. Therefore, despite some uncertainty in the estimates as a result of using expenditure‐based multipliers, this type of analysis can still differentiate between high and low emitters. Consequently, emission estimates generated using expenditure‐based carbon multipliers allow for useful assessments of emissions, including where they can be reduced and redistributed.

Overall, we generate carbon multipliers for 307 COICOP categories, each of which can be further categorized into 12 broad COICOP spend categories (e.g., Transport, Food, Housing, etc.; for the full list see Supporting Information S6, Table SI2).

### Calculation of carbon multipliers by TCS category (TCS‐COICOP mapping)

Next, as spend data is on the TCS level, we need to generate carbon multipliers for the 252 TCS categories using the carbon multipliers established for the 307 COICOP categories.

For each TCS category, we review available COICOP categories to identify the corresponding COICOP categories. In cases where there is a simple one‐to‐one mapping, we simply carry over the available carbon multiplier directly. For example, the TCS category “Internet” maps directly to the COICOP category “Internet subscription fees,” which carries a carbon multiplier of 0.17.

For those TCS categories where multiple corresponding COICOP categories exist, we construct a household spend‐weighted average of the corresponding multipliers. For example, for TCS category “Gas & Electricity,” COICOP categories “Gas” and “Electricity” are available with multipliers 6.966 and 2.249, respectively. According to the LCFS, 54.3% of the combined spend in these categories is spent on electricity, we therefore construct a spend‐weighted multiplier average of 4.405 for the TCS category of “Gas & Electricity.”

In addition to calculating the relevant carbon multipliers, we determine the most relevant broad COICOP category for each TCS category (see Supporting Information S5 Table SI1). Supporting Information S7 (Table SI3) lists the five TCS categories with the highest carbon multipliers. Supporting Information S8 (Table SI4) contains the entire TCS‐COICOP mapping table.

Out of 252 TCS categories, 55 were out of scope of the COICOP classification system as they are not included in the definition of household expenditure in the United Kingdom (Office for National Statistics, 2020) and are also not part of the carbon profiles estimated from the LCFS survey. These non‐consumption expenditure categories include financial investments, loan, mortgage, council tax, charity, tax, and duty free spend (see Supporting Information S8, Table SI4), representing 26% of spend in the LBG sample.

After excluding these categories, ATM withdrawal constitutes 14% of overall spend in our sample. Drawing on information from the UK Finance's 2020 report on money spending habits in the United Kingdom (UK Finance, 2020), we use the mapping approach described above to determine the carbon multipliers associated with the top 20 cash spend categories (see Supporting Information S9, Table SI5) and corresponding cash spend proportions. More information on the steps involved in this calculation can be found in the SI.

### Calculation of carbon footprints for the LBG sample and the LCFS sample

We can now calculate TCS‐level emissions (kg CO_2_e) for each individual in the sample by multiplying TCS‐level spend data (£) with TCS‐level carbon multipliers (kg CO_2_e/£). Using COICOP‐level data, we repeat the parallel exercise for the LCFS sample (*N* = 5473).

For the LBG transaction data, we sum TCS and ATM‐related emissions by the corresponding broad COICOP category to calculate domain‐specific emissions by individual. This allows us to compare LBG and LCFS emission estimates at the broad COICOP category level. Finally, by summing these for each individual, we derive total annual emission figures.

Supporting Information S4 (Figure SI1) summarizes the steps described throughout this section.

## QUANTITATIVE COMPARISON

The focus of our analysis is a quantitative comparison of carbon emission distributions derived from transaction data and LCFS across broad COICOP categories (e.g., housing, transport, food), and key demographic variables (age, income, region). To ensure a like‐for‐like comparison, we compare “sole account” holders in the LBG sample (*N* = 51,000) to “individuals” in LCFS (equivalized estimates); and separately, “joint account” holders in the LBG sample (*N* = 50,744) to “households” in LCFS. Supporting Information S10 (Table SI6) provides a summary of these distinctions.

To quantify the degree of overlap between corresponding distributions, we report the Kolmogorov–Smirnov ($KS$) statistics (for more detail see the SI). The $KS$ statistic is bounded between 0 and 1, where the two extremes indicate no overlap and a perfect overlap, respectively. As a useful reference point, we note that the value of the KS statistic when comparing the emission distributions from the top and the bottom income deciles in the LCFS sample is 0.60.

### Emission groups: Overall, housing, transport, food

#### Overall emissions distributions

**FIGURE 1:**
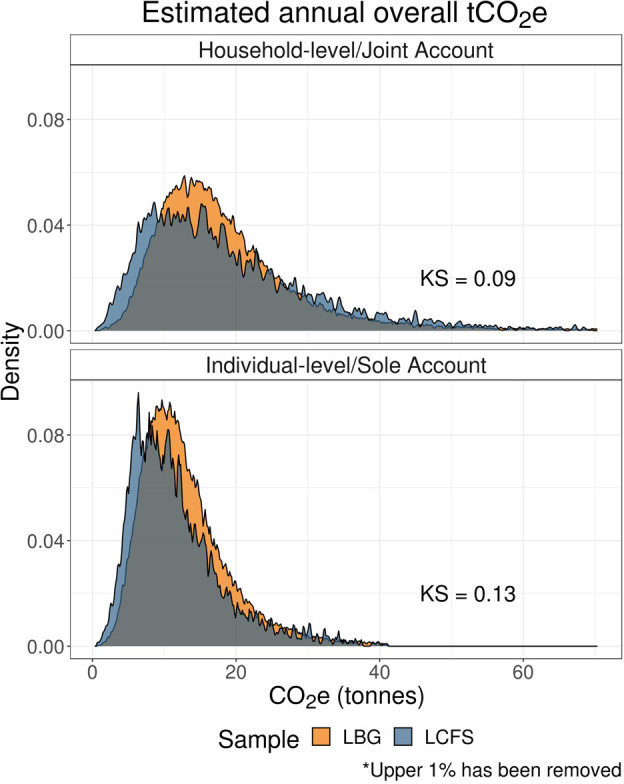
Distributions of overall carbon footprint estimates. Low‐resolution data underlying this figure are available in Supporting Information S13 (Table SI9).

Figure 1 shows the distributions of overall annual footprint estimates by sample (LBG vs. LCFS) and estimate type (Household/Joint vs. Individual/Sole account). Each figure reports the $KS$ statistics.

We see a high degree of overlap between these distributions, as evidenced by the low KS statistics. Average household emissions from the LCFS are only slightly higher than the average household emissions from the LBG sample (${{\rm{M}}_{{\rm{LCFS}}}} = {\rm{\;}}19.78,{\rm{\;\;}}95{\rm{\% \;CI\;}}[ {19.37,{\rm{\;}}20.15} ]$; ${{\rm{M}}_{{\rm{LBG}}}} = {\rm{\;}}19.19,{\rm{\;\;}}95{\rm{\% \;CI\;}}[ {19.06,{\rm{\;}}19.31} ]$). Conversely, for individual‐level carbon emissions, we find that the average LCFS emissions estimates are lower than the average LBG estimates (${{\rm{M}}_{{\rm{LCFS}}}} = {\rm{\;}}12.28,{\rm{\;\;}}95{\rm{\% \;CI\;}}[ {12.05,{\rm{\;}}12.46} ]$; ${{\rm{M}}_{{\rm{LBG}}}} = {\rm{\;}}13.29,{\rm{\;\;}}95{\rm{\% \;CI\;}}[ {13.20,{\rm{\;}}13.36} ]$).

These results are consistent with a scenario where (1) LBG transaction data does not capture the entirety of household and individual‐level expenditure, and (2) LBG sole account spending also captures some proportion of household spending. The effect of the former is minimized from the sample selection (see Section 2.2.1), and therefore the latter is likely to impact our estimates more. Notably, a significant proportion of households do not hold a joint account (Money Saving Expert, 2019).

Next, we examine the emission distributions for the top three broad COICOP categories. Descriptive statistics for all emissions groups are reported in Supporting Information S11 (Table SI7), while emission shares by emission category, sample, and estimate types are reported in Supporting Information S12 (Table SI8).

#### Housing emissions distributions

Housing‐related emissions primarily capture emissions arising from spend on gas, electricity, and water charges, constituting about 33–37% of overall carbon footprints across samples.

**FIGURE 2:**
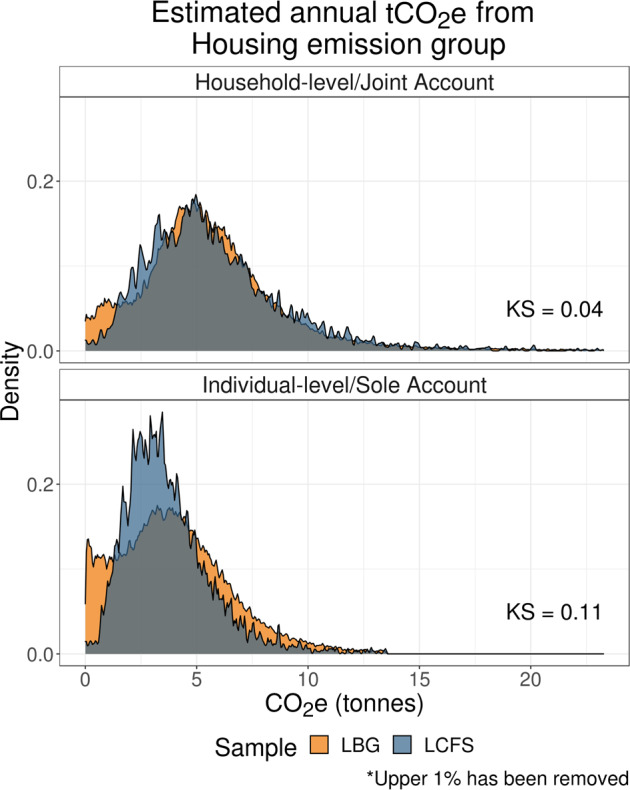
Distributions of housing‐related carbon footprint estimates. Low‐resolution data underlying this figure are available in Supporting Information S13 (Table SI9).

While Figure 2 shows a high level of correspondence between household‐level and joint account housing‐related estimates, we see larger differences between individual‐level and sole account emission distributions.

First, we see that low emission estimates are more common in the LBG sample. This might stem from the fact that a relatively high proportion of the sole account sample shows evidence for “pay as you go” energy payments. These prepaid energy meters are often preferred by landlords, and it is possible that there are relatively more renters in the sole account sample. Compared to monthly direct debit, prepaid energy payments are more likely to be spread across multiple accounts within the household or paid by cash, which could explain why we see a higher proportion of individuals with relatively low energy payments in the LBG sample.

Second, we also note that a higher share of the LBG sole account sample has relatively high housing‐related emission estimates (above 5 tonnes of CO_2_e). As discussed in Section 3.1.1, this pattern is consistent with the assumption that some proportion of the sole account sample captures household‐level spend.

#### Transport emissions distribution

Transport‐related estimates include emissions originating from spend on all modes of transport, including petrol, air and rail travel, public transport, and car finance. It constitutes about 24–26% of overall household and individual‐level emissions across samples.

**FIGURE 3:**
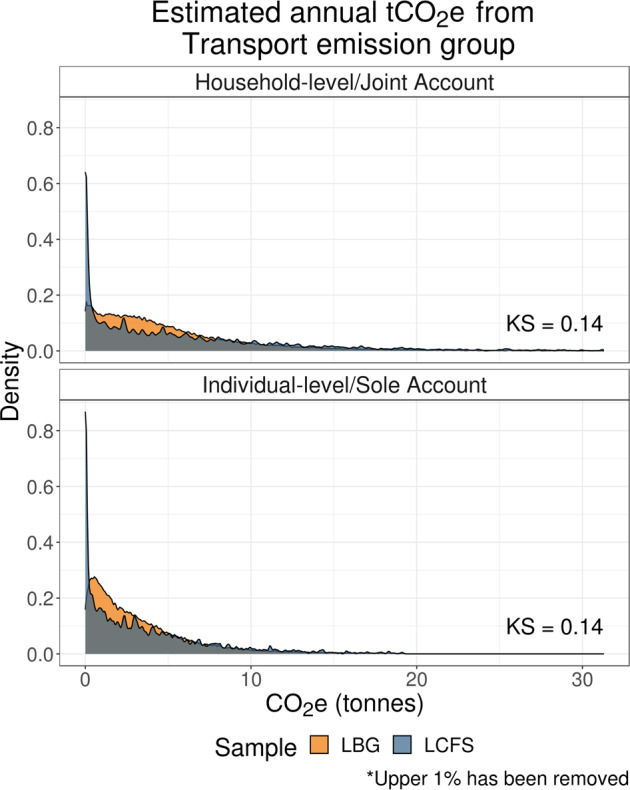
Distributions of transport‐related carbon footprint estimates. Low‐resolution data underlying this figure are available in Supporting Information S13 (Table SI9).

As Figure 3 and Supporting Information S11 (Table SI7) demonstrate, although the medians of transport‐related emission estimates show a reasonable degree of correspondence, the average LCFS estimates are higher than LBG estimates.

This difference may result mechanically from how spend data is collected in the LCFS. Specifically, annual petrol spend estimates are extrapolated from the shorter, 2‐week spend diary used by the LCFS (see SI). This may fail to capture more infrequent petrol spending occurring outside of this critical 2‐week period and overestimate spend for those who did purchase petrol during this period.

This feature of the LCFS approach could also explain why LCFS transport‐related emission distribution peak at zero, and have a long tail. The LBG distribution may, in fact, be a more truthful approximation of transport‐related carbon emissions.

#### Food emissions distributions

Food‐related LBG estimates are calculated as emissions arising from general supermarket spend, while for the LCFS sample, they are calculated from all spend on specific foodstuff and non‐alcoholic drinks consumed at home. These constitute about 15–18% of overall emissions across samples.

Using general supermarket spending to proxy specific food spending is a crude but necessary assumption, given the lack of basket‐level information available in financial transactions. Supermarket spending figures include expenditure on both food and non‐food items available. Similarly, carbon multipliers for supermarket‐related TCS categories reflect a weighted average of relevant food and non‐food COICOP categories. As a result, LBG food emission estimates will necessarily exceed LCFS estimates that are restricted to food‐related COICOP categories.

**FIGURE 4:**
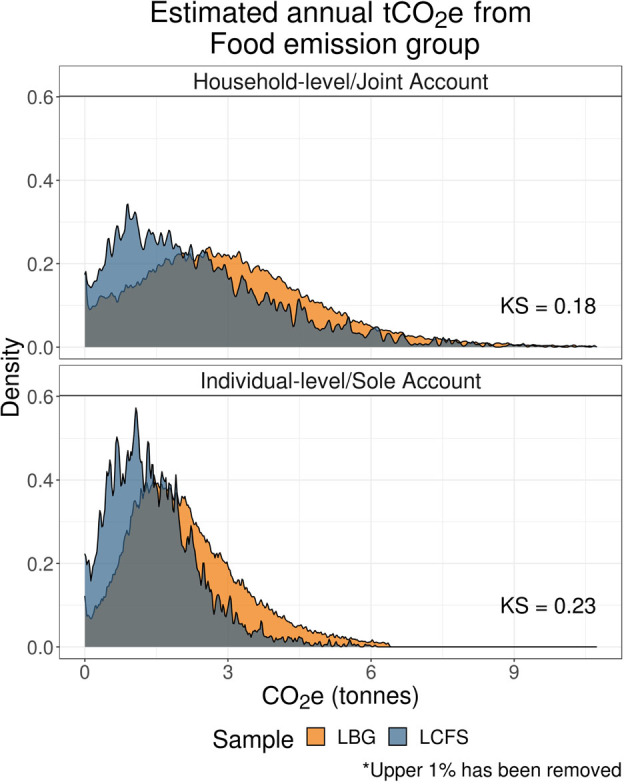
Distributions of food‐related carbon footprint estimates. Low‐resolution data underlying this figure are available in Supporting Information S13 (Table SI9).

As expected, average food‐related emission estimates are significantly higher in the LBG sample than in the LCFS sample, as shown by Figure 4 and Supporting Information S11 (Table SI7). In Section 4, we will return to the challenge associated with a lack of basket‐level information in financial transaction data.

### Demographics: Age, income, region

For climate policy insights, it is useful to break down emissions by demographic characteristics (Owen & Barrett, 2020). We now compare the overlap between LCFS and LBG overall carbon footprint distributions across different levels of age, income, and region to demonstrate that transaction‐based emission estimates represent a valuable source of insights. We note that the LCFS emission distributions show more volatility due to the relatively small sample sizes in different demographic categories.

#### Age

**FIGURE 5:**
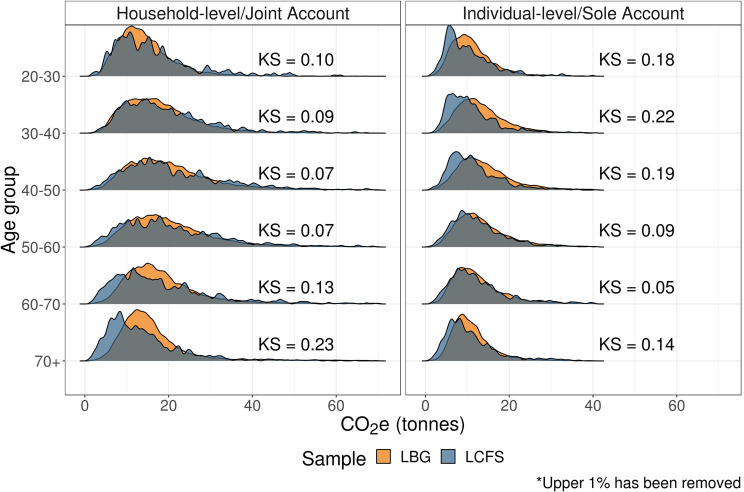
Carbon footprint distributions by age group. Low‐resolution data underlying this figure are available in Supporting Information S13 (Table SI9).

Figure 5 shows overall emission distributions across six different age groups. Both the LBG and LCFS distributions suggest an inverse U‐shaped relationship between emissions and age groups, where emissions peak in the 40–50 and 50–60 age groups.

In line with the assumption that some proportion of the LBG sole account sample captures household‐level spend, we see consistently higher LBG estimates in the age groups where individuals are most likely to live in a multi‐person household (20–50 age groups).

Interestingly, we also see a slight divergence between household‐level estimates in older age groups (60+), where LCFS estimates are generally lower, driven by differences in transport and restaurant‐related emission groups. It is possible that the LCFS data collection methodology fails to adequately capture the relatively higher emission share of non‐regular travel and hotel spend in this age group.

#### Income

**FIGURE 6:**
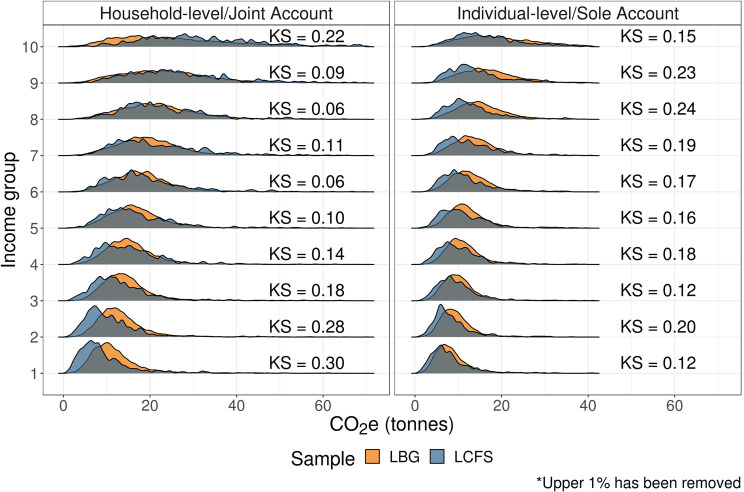
Carbon footprint distributions by income group. Low‐resolution data underlying this figure are available in Supporting Information S13 (Table SI9).

Figure 6 shows overall carbon footprint distributions across income groups, where groups 1 and 10 correspond to the lowest and highest income groups, respectively. For both household‐ and individual‐level estimates, we see a strong positive relationship between income and emissions.

However, for household‐level estimates, we observe some divergence: in the highest income group, LCFS estimates exceed LBG, whereas in the lower income groups, LBG slightly exceed LCFS. Again, this is partly driven by corresponding differences in transport‐related emissions between the two samples.

#### Region

**FIGURE 7:**
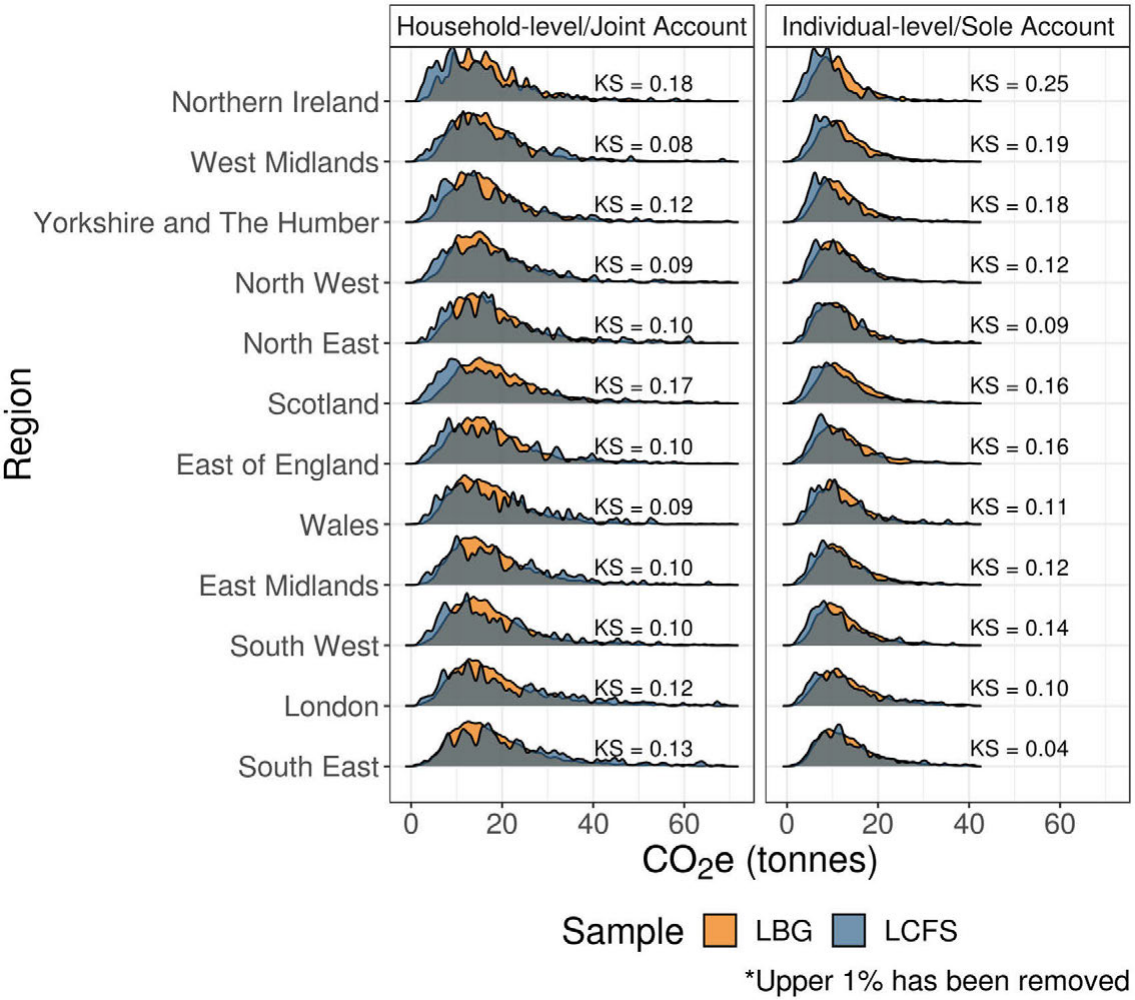
Carbon footprint distributions by region. Low‐resolution data underlying this figure are available in Supporting Information S13 (Table SI9).

Finally, Figure 7 shows overall emission distributions across regions in the United Kingdom. Again, we see generally better alignment for households. We observe the largest differences for regions with the highest (London, South East) and lowest (Northern Ireland, Scotland) average incomes.

### Summary of quantitative sample comparisons

Comparing the carbon footprint distributions derived from LBG transaction data with those from the LCFS household survey, we find broad agreement between the two data sources across key emission areas and demographic characteristics.

Where the distributions differ, we identify methodological differences in how spend is captured. These include prepaid utilities potentially paid by cash (see housing‐related emissions), a 2‐week spend diary not capturing all fuel spend (transport), and lack of basket‐level information in transaction data (food). We also observe some differences where “sole accounts” potentially capture spend for multi‐person households, rather than just a single person.

A future study could explore the similarities and differences between carbon emission estimates from financial transactions and those from an activity‐based carbon calculator capturing physical quantities.

## QUALITATIVE COMPARISON

We now explore a broader, qualitative comparison of the advantages and disadvantages of using transactions versus household surveys and carbon footprint calculators. As described in Table Table 1, this comparison considers a range of dimensions, including data availability, quality, and detail.

**TABLE 1:** Overview of strengths and weaknesses of carbon footprint data sources

### Data availability

Based on their relative availability, only household spend surveys are currently publicly available of the three data sources. Physical activity surveys require specific data to be collected and then made available, and transaction data, like other data assets generated by commercial activity, are subject to data access constraints and data privacy protection. We recognize this represents a major barrier to benefiting from transaction data to support climate policy and return to this topic in Section 5.

#### Big data, scalability, representative sample

Assuming data access is available, transactional data‐based carbon measurements could in principle be constructed for the majority of the adult population in developed economies. This is a significant advantage over surveys and physical carbon footprint calculators, which rely on active participation.

While both transaction and survey data allow for a representative sample (the former by data volumes, the latter by survey design), those using physical carbon footprint calculators are almost certainly self‐selecting to take part which may bias results (e.g., they are likely to be individuals who are more conscious of their carbon footprint; West et al., 2016).

Also, since transactional data captures the behavior of a significant proportion of the population, it is ideally suited to address a key limitation of survey‐based methods by identifying and modeling the behavior of the upper 1% of the income distribution.

#### Longitudinal view, frequency of data points

Household surveys and physical carbon calculators only provide a point‐in‐time snapshot of carbon emissions. In contrast, transactional data allows for measurement over time, making it ideally suited for analyzing the effects of policy changes. Furthermore, the high frequency of transaction data supports much more regular carbon profiling, unlike infrequent survey data collection that may result in biased spending estimates (see Section 3.1.3).

#### Demographic variables

Carbon footprint data combined with demographic variables (e.g., age, income, and region) enables analyses of subgroup effects. Household surveys and transaction data already include this information; however, carbon calculators are not a feasible data source due to sample selection effects.

### Data quality

#### Completeness

Surveys can provide a detailed, comprehensive picture of a household's spending profile. In contrast, 22% of UK adults hold accounts across several financial service providers (Moon et al., 2015). Therefore, using transaction data from just one provider may underestimate true expenditure.

Open Banking technology could address this completeness problem, providing a complete view of spending activity across multiple providers (Open Banking Implementation Entity, 2022). But these solutions still require explicit customer opt‐ins and active participation which reduces sample sizes.

Physical carbon calculators are also limited by the number of survey questions being posed and it is recognized that there is a trade‐off between complexity and usability (West et al., 2016).

#### Reliability

Household surveys and carbon footprint calculators rely on self‐reported data, whereas transactions represent an objective view of an individual's spending behavior, capturing actual outgoing flows.

### Data detail

#### Granularity

A key limitation of using financial transactions when compared to survey methods is the lack of basket‐level information. For example, as discussed in Section 3.1.4, whilst transactions provide the total amount of spending in a supermarket, the types and quantities of food items such as beef or vegetables are unknown.

A related point concerns cash spending, where financial transactions cannot identify categories of spending (see Section 3.1.2). We discuss the granularity issue further in Section 4.4.

#### Physical volumes

Physical carbon calculators capture volumes directly for selected emission categories. Whereas carbon multipliers used by spend data‐based methods are constructed from a monetary input–output system and rely on spending as a proxy for volume. This is a limitation as some spend differences will inevitably reflect price differences rather than volume differences.

This so‐called “Proportionality Assumption Uncertainty” (Lenzen, 2000, p. 138) remains an issue across all footprint calculation methods which rely on CO_2_e/spend‐based multipliers, including transaction and survey data‐based estimates.

#### Differentiation

Neither the COICOP categories used for LCFS and LBG calculations, nor physical carbon footprint calculators are sufficiently granular to recognize more subtle differences within the same product category. These differences may include brands or slight variations of products.

These types of errors can be characterized as aggregation and allocation uncertainties (Lenzen, 2000). Efforts to accurately capture emissions associated with the entire life cycle of a particular product (Meinrenken et al., 2020) will prove useful in addressing this problem.

### Summary of qualitative comparison

In summary, whilst these data sources have different strengths and weaknesses, the transaction data approach offers the most advantages (see Table Table 1). A comprehensive solution could build on transactional data whilst addressing some of its methodological shortcomings, in particular, concerns around data access and new innovations to improve data completeness and data granularity.

We return to a discussion on data access in Section 5. On challenges related to data completeness and lack of basket‐level information, one consumer app (Evocco, 2022) addresses these issues directly by providing a service to scan food shopping receipts. Whilst this is highly innovative, the approach still requires active participation and is therefore subject to similar criticisms as surveys, for example, low sample sizes.

Alternative innovations are needed to address these key methodological issues. A promising direction is to develop hybrid approaches for calculating carbon multipliers. These could incorporate additional information from population segments or product supply chains to improve data granularity, and statistical techniques to infer missing data and help improve data quality.

## CONCLUSION

In this study, we provide a detailed overview of our approach toward calculating carbon footprints from transaction data, and quantitatively compare our emission estimates to those obtained from the LCFS household spend survey, finding broad correspondence between emissions estimated from the two data sources. Additionally, we provide a detailed qualitative comparison of the advantages and disadvantages of using transactions versus alternative data sources. Based on these analyses, we conclude that transaction data offers a valuable additional data source for climate policy makers, providing key advantages over survey‐based data sources including detailed and objective measurements, larger sample sizes, and longitudinal, frequent data capture.

Transaction data also promises to provide individuals, businesses, and other organizations easier access to their own detailed carbon footprint profiles, without the frictions imposed by complex survey questionnaires or expensive engagements with third‐party providers.

However, the restricted data availability of financial transactions remains a key challenge to realizing these benefits. Transactions are stored by banks and other commercial organizations, and are therefore confidential and protected. New data sharing solutions and cultural norms are required to bring these resources safely and securely to policy makers and other concerned parties.

We see two broad routes forward. The first is via personal data donation. With the introduction of new General Data Protection Regulation (European Union, 2016), the general public can now access data collected about them by organizations. With the additional ease of access introduced with standards of Open Banking technology (Open Banking Implementation Entity, 2022), financial transactions can—in principle—be donated by the public at a scale that is useful to carbon policy.

Recent research finds over half of those individuals surveyed would be willing to donate their data to benefit society (Skatova & Goulding, 2019). However, in reality, only 30% of users may choose to share their anonymized carbon footprint data for research purposes (Andersson, 2020). In practice, new, dedicated services would also be required to manage data sharing and the underlying complex logistics—and these must be funded, incentivized, and developed at scale.

The second route focuses instead on commercial data donation. With a number of banks and credit card providers starting to introduce new carbon tracking features into digital banking (Gausden, 2021) and dedicated start‐ups developing new carbon apps and calculators, these organizations should recognize a social responsibility to make carbon insights more widely available.

This cultural shift toward sharing carbon data need not jeopardize consumer data privacy. With appropriate data anonymization and aggregation practices, standards can be safely maintained. The formatting for disclosures could follow existing standards (UK Data Service, 2021) and regular reporting frequencies could be established, paralleling quarterly business performance disclosures.

As carbon profiling from transactions inevitably becomes much more commonplace across the financial services industry, a commensurate culture of data sharing and transparency is also required to ensure benefits are fully realized by policymakers, customers, and other key stakeholders.

## Supplementary Information

**
Supporting Information S4**: This supporting information provides an overview of the steps involved in our methodology (Figure SI1).

**Supporting Information S5**: This supporting information lists the relevant TCS categories and their COICOP broad group mapping (Table SI1).

**Supporting Information S6**: This supporting information lists the 12 broad COICOP categories (Table SI2).

**Supporting Information S7**: This supporting information lists the 5 TCS categories with the highest carbon multipliers (Table SI3).

**
Supporting Information S1**: This supporting information provides a description of the datasets used for the calculation of carbon multipliers.

**
Supporting Information S2**: This supporting information provides a description of the calculation of carbon multipliers and cash spend proportions for cash spend categories.

**
Supporting Information S3**: This supporting information provides an overview of the Kolmogorov‐Smirnov statistics.

**
Supporting Information S8**: This supporting information provides the TCS‐COICOP mapping table (Table SI4).

**
Supporting Information S9**: This supporting information provides the ATM‐COICOP mapping table (Table SI5).

**
Supporting Information S10**: This supporting information provides information on the datasets used in the validation (Table SI6).

**
Supporting Information S11**: This supporting information provides carbon footprint descriptive statistics by broad COICOP category (Table SI7).

**
Supporting Information S12**: This supporting information provides information on the broad COICOP category average overall emission shares (Table SI8).

**Supporting Information S13**: This supporting information contains low‐resolution data underlying the figures presented in the manuscript (Table SI9).

## Data Availability

The data that support the findings of this study are available from LBG but restrictions apply to the availability of these data, which were used under license for the current study, and so are not publicly available. Data are available from the authors upon reasonable request and with permission of LBG.

## Footnotes

1 The OECD equivalence scale reference case (weight = 1) is a two‐adult household with no children. Each household in the LCFS is assigned an equivalence factor. Households with a single adult score 0.67, each subsequent adult adds 0.33, and children under the age of 14 score 0.2. For example, a household of two adults and two children scores 1.4. Income and expenditure is first divided by the recorded equivalence factor so that each entry is equivalent to a household of two adults and no children. Finally, the entries are further multiplied by 0.67 to convert to a solo adult.

2 It is standard practice within industry for banks to maintain internal, proprietary Transaction Categorization Systems (TCS). This system underpins several crucial analytical processes and is continuously improved using a variety of private and public sources.
