# Supplementary material for: Estimating carbon footprints from large scale financial transaction data
Source: J Ind Ecol. 2022 Dec 27;27(1):56–70. doi: 10.1111/jiec.13351 (PMC13090182; doi:10.1111/jiec.13351)
Supplement: Supplementary file 1 — Supporting Information S4: This supporting information provides an overview of the steps involved in our methodology (Figure SI1). [file 44498_2023_2701005_MOESM1_ESM.docx]

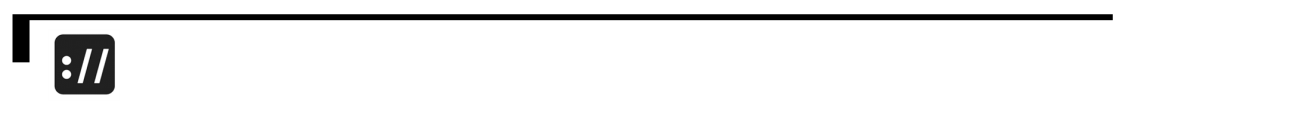


SUPPORTING INFORMATION FOR:

Trendl, A., Owen, A., Vomfell, L., Kilian, L., Gathergood, J., Stewart, N. & Leake, D. (2022.) Estimating carbon footprints from large scale financial transaction data. *Journal of Industrial Ecology.*


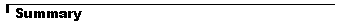


This supporting information provides a description of the datasets used for the calculation of carbon multipliers.


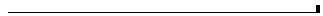


**Carbon multiplier data sources**

# COICOP

In our methodology, we combine carbon emission and household spend data for the same set of consumption categories to calculate carbon multipliers. The shared consumption categorisation system is COICOP (Classification of Individual Consumption by Purpose; United Nations, 2014) – an international framework for the standardised classification of household expenditure across countries. This system is used in various key economic indicators, including customer prices indices (CPI) and gross domestic product, allowing for important cross-country comparisons (United Nations, 2018).

# Consumption-based GHG emissions by COICOP

The input-output models underpinning the process of estimating the UK’s GHG emissions are constructed from observed economic data and capture the interrelationships between industries that consume goods (inputs) from other industries in the process of making their own products (outputs; Miller & Blair, 2009). The fundamental Leontief equation, $\mathbf{x=}\left( \mathbf{I-A} \right)^{\boldsymbol{-1}}\mathbf{y}$, indicates the inter-industry requirements of each sector to deliver a unit of output to final demand. Since the 1960s, the input-output framework has been extended^[[1]](#footnote-2)^ to account for increases in the pollution associated with industrial production due to a change in final demand. For detailed discussion of the UKMRIO database including additional notes on construction and application see Barrett et al., (2013); Hardt et al., (2018); Lenzen et al., (2010); Owen et al., (2018); Sakai et al., (2017); Scott et al., (2018); Wiedmann, (2009); Wiedmann et al., (2010); and Wiedmann & Barrett, (2013).

The “consumption-based” approach used in UKMRIO is one of three available calculation approaches, the others being “territorial” or “production” based. A territorial-based methodology takes into account emissions occurring within the territory of the country, whereas a production-based approach takes into account the emissions associated with residents and businesses registered within a country, regardless of where the emissions geographically occur (UK Government, 2021). In contrast, a consumption-based approach measures emissions associated with the entire supply chain of goods and services available within the economy, irrespective of where those emissions occurred, and assigns these to the final consumer. In the UK, consumption-based estimates exceed territorial and production-based estimates, and offer a more complete picture of the total GHG emissions by accounting for emissions arising throughout the entire lifecycle of the goods and services consumed.

Crucially, in the UKMRIO, Supply and Use Tables split household final demand spend into COICOP categories, meaning it is possible to calculate household footprint by COICOP product. This COICOP categorisation also applies to spend data, our second key data source used to produce carbon multipliers.

# Living Costs and Food Survey (LCFS): household and individual-level spend by COICOP

Since 1957, the Office for Nationals Statistics (ONS) has surveyed UK households annually on their weekly expenditure (Office for National Statistics, 2017). In 2008, this survey became known as the Living Costs and Food Survey (LCFS). Annually, approximately 5,500 households take part in the survey, which contains a demographic questionnaire followed by a detailed survey of household expenditure (Office for National Statistics, 2020). Each member aged over 16 of a selected household is asked to keep a detailed spend diary for two weeks (Bulman et al., 2017), and responses are collected all year long. The survey is weighted to account for non-response bias and to create a representative sample of the population by age, gender, and region.

The LCFS is used to provide information on retail price indices, National Account estimates of household expenditure, the effect of taxes and benefits, and trends in nutrition. As well as providing information on household spend across COICOP categories (more than 300 different product types), additional information is collected such as the composition, age, sex and occupation of household members; total household income; and the household’s location, tenure, and dwelling type.

While the LCFS collects household-level spend data, it is also possible to use this data to calculate individual-level spend estimates. To generate individual spend profiles from household-level data, we use a process known as equivalisation to obtain equivalised, one-person household-level estimates (Gough et al., 2011). A modified OECD equivalence scale, as preferred by UK Government, is applied to convert each survey entry into the equivalent spends if the household was a single adult^[[2]](#footnote-3)^. We used household and one person household-level spend estimates (N = 5473) from the 2018 LCFS. For these respondents, we also obtained data on income group (1-10); each UK NUTS 2 region; and age of the household reference person.

# Carbon multipliers by COICOP

To create a carbon multiplier by COICOP category, then, we combined 2018 estimates of GHG emissions by COICOP category (Ktonnes CO2e, obtained from the EE-MRIO model) with household spend estimates for the same set of COICOP categories (£ millions per year) from the 2018 LCFS (covering the period between April 2018 and March 2019 ). Carbon multipliers by COICOP category (kg CO2e/£) are calculated by dividing emissions with the relevant household spend figure. Creating a standardised measure of the GHG emissions associated with £1 spend based on the purpose of that spend allowed us to easily combine these multipliers with spend data by COICOP category and calculate household-level footprints.

Overall, we generated carbon multipliers for 307 COICOP categories, each of which can be further categorised into 13 broad spend divisions (e.g., Transport, Food, Housing, etc.). We used a shortened version of COICOP with only 12 broad categories by merging categories 12 (Insurance and Financial Services) and 13 (Personal care, social protection and miscellaneous goods and services) into one category (Miscellaneous goods and services).

**References**

Barrett, J., Peters, G. P., Wiedmann, T., Scott, K., Lenzen, M., Roelich, K., & Le Quéré, C. (2013). Consumption-based GHG emission accounting: a UK case study. *Climate Policy*, *13*(4), 451–470. https://doi.org/10.1080/14693062.2013.788858

Bulman, J., Davies, R., & Carrel, O. (2017). *Living Costs and Food Survey - Technical Report for survey year: 2015-2016*. https://www.ons.gov.uk/file?uri=/peoplepopulationandcommunity/personalandhouseholdfinances/incomeandwealth/methodologies/livingcostsandfoodsurvey/livingcostsfoodtechnicalreport2015.pdf

Gough, I., Abdallah, S., & Johnson, V. (2011). The distribution of total greenhouse gas emissions by households in the UK, and some implications for social policy. In *Centre for Analysis of Social Inclusion* (Vol. 152). https://sticerd.lse.ac.uk/case/_new/publications/abstract/?index=3863

Hardt, L., Owen, A., Brockway, P., Heun, M. K., Barrett, J., Taylor, P., & Foxon, T. J. (2018). Untangling the drivers of energy reduction in the UK productive sectors: Efficiency or offshoring? *Applied Energy*, *223*, 124–133. https://doi.org/https://doi.org/10.1016/j.apenergy.2018.03.127

Lenzen, M., Wood, R., & Wiedmann, T. (2010). Uncertainty Analysis for Multi-Region Input–Output Models – a Case Study of the UK’s Carbon Footprint. *Economic Systems Research*, *22*(1), 43–63. https://doi.org/https://doi.org/10.1080/09535311003661226

Miller, R. E., & Blair, P. D. (2009). *Input-output analysis: foundations and extensions* (2nd ed.). Cambridge University Press.

Office for National Statistics. (2017). *Living Costs and Food Survey*. https://www.ons.gov.uk/peoplepopulationandcommunity/personalandhouseholdfinances/incomeandwealth/methodologies/livingcostsandfoodsurvey

Office for National Statistics. (2020). *Living Costs and Food Survey technical report: financial years ending March 2018 and March 2019 - Office for National Statistics*. https://www.ons.gov.uk/peoplepopulationandcommunity/personalandhouseholdfinances/expenditure/methodologies/livingcostsandfoodsurveytechnicalreportfinancialyearsendingmarch2018andmarch2019

Owen, A., Scott, K., & Barrett, J. (2018). Identifying critical supply chains and final products: An input-output approach to exploring the energy-water-food nexus. *Applied Energy*, *210*, 632–642. https://doi.org/https://doi.org/10.1016/j.apenergy.2017.09.069

Sakai, M., Owen, A., & Barrett, J. (2017). The UK’s emissions and employment footprints: Exploring the trade-offs. *Sustainability*, *9*(7). https://doi.org/10.3390/su9071242

Scott, K., Giesekam, J., Barrett, J., & Owen, A. (2018). Bridging the climate mitigation gap with economy-wide material productivity. *Journal of Industrial Ecology*, *23*(4), 918–931. https://doi.org/https://doi.org/10.1111/jiec.12831

UK Government. (2021). *UK’s Carbon Footprint 1997 - 2018*. https://assets.publishing.service.gov.uk/government/uploads/system/uploads/attachment_data/file/979588/Defra_UK_carbon_footprint_accessible_rev2_final.pdf

United Nations. (2014). *COICOP: Detailed structure and explanatory notes*. http://unstats.un.org/unsd/cr/registry/regcst.asp?Cl=5

United Nations. (2018). *Classification of Individual Consumption According to Purpose (COICOP) 2018*. https://unstats.un.org/unsd/classifications/unsdclassifications/COICOP_2018_-_pre-edited_white_cover_version_-_2018-12-26.pdf

Wiedmann, T. (2009). A review of recent multi-region input–output models used for consumption-based emission and resource accounting. *Ecological Economics*, *69*(2), 211–222. https://doi.org/10.1016/J.ECOLECON.2009.08.026

Wiedmann, T., & Barrett, J. (2013). Policy-relevant applications of environmentally extended MRIO databases – Experiences from the UK. *Economic Systems Research*, *25*(1), 143–156. https://doi.org/10.1080/09535314.2012.761596

Wiedmann, T., Wood, R., Minx, J., Lenzen, M., Guan, D., & Harris, R. (2010). A Carbon Footprint Time Series of the UK – Results From a Multi-Region Input–Output Model. *Economic Systems Research*, *22*(1), 19–42. https://doi.org/https://doi.org/10.1080/09535311003612591

1. If $\mathbf{f}$ is a row vector of annual emissions emitted by each industrial sector let $\mathbf{e= f}{\hat{\mathbf{x}}}^{\mathbf{-1}}$**.** Then, $\mathbf{ex = e}\left( \mathbf{I-A} \right)^{\boldsymbol{-1}}\mathbf{y}$, which simplifies to $\mathbf{F = eLy}$ where $\mathbf{F}$ is the emissions emitted to meet final demand $\mathbf{y}$ [↑](#footnote-ref-2)
2. The OECD equivalence scale reference case (weight =1) is a two-adult household with no children. Each household in the LCFS is assigned an equivalence factor. Households with a single adult score 0.67, each subsequent adult adds 0.33, and children under the age of 14 score 0.2. For example, a household of 2 adults and 2 children scores 1.4. Income and expenditure is first divided by the recorded equivalence factor so that each survey is equivalent to a household of 2 adults and no children, then the entries are further multiplied by 0.67 to convert to a solo adult. [↑](#footnote-ref-3)
