# Supplementary material for: Estimating carbon footprints from large scale financial transaction data
Source: J Ind Ecol. 2022 Dec 27;27(1):56–70. doi: 10.1111/jiec.13351 (PMC13090182; doi:10.1111/jiec.13351)
Supplement: Supplementary file 2 — Supporting Information S5: This supporting information lists the relevant TCS categories and their COICOP broad group mapping (Table SI1). [file 44498_2023_2701005_MOESM2_ESM.docx]

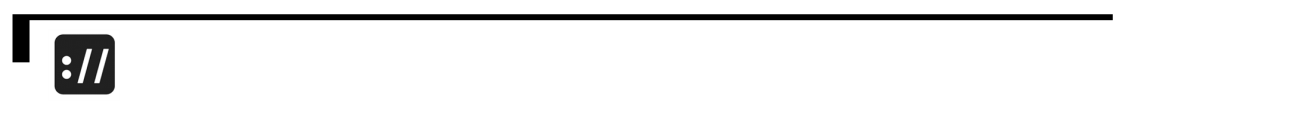


SUPPORTING INFORMATION FOR:

Trendl, A., Owen, A., Vomfell, L., Kilian, L., Gathergood, J., Stewart, N. & Leake, D. (2022.) Estimating carbon footprints from large scale financial transaction data. *Journal of Industrial Ecology.*


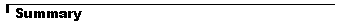


This supporting information provides a description of the calculation of carbon multipliers and cash spend proportions for cash spend categories.


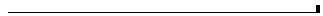


**ATM spend carbon multipliers and spend proportions**

To approximate the carbon footprint associated with cash spend, we drew on information from the UK Finance’s 2020 report on money spending habits in the UK (UK Finance, 2020, Section 3, Table 15.2). This report provides information on the amount of cash spent in the UK in 52 spend categories. We selected the top 20 spend categories (by restricting the list to categories with higher than 1% share of overall cash spent in the UK), and determined carbon multipliers for each of these categories by separately mapping them to the COICOP categories and calculating the household-spend weighted average of the relevant COICOP multipliers.

Because we only mapped categories that cover more than 1% of cash spend, and these selected categories cumulatively only cover 90.47% of cash spend, we then re-normalised the amount of cash associated with each of these categories so that we account for cash spend in its entirety. For example, if about 10% of overall cash spend is spent in Restaurants, then the re-normalised cash spend share of Restaurants will be 11%. Finally, we determined the most relevant broad COICOP category for each of these 20 ATM spend categories**. Table SI_2_ATM_mapping_1** shows the renormalized cash spend shares aggregated by broad COICOP category, while **Table SI5** shows the mapping between the 20 ATM and COICOP categories.

Table SI_2_ATM_mapping_1: *Cash spend by COICOP broad category*

| **COICOP broad category number** | **COICOP broad category name** | **Cash spend percentage** |
| --- | --- | --- |
| 1 | Food | 29.9% |
| 11 | Restaurants & Hotels | 23.8% |
| 12 | Miscellaneous | 15.7% |
| 5 | Furnishings | 8.8% |
| 9 | Recreation | 8.2% |
| 7 | Transport | 6.3% |
| 3 | Clothing | 3.2% |
| 0 | Non-consumption category | 2.6% |
| 6 | Health | 1.4% |

Next, for each individual in the sample, we apportioned their 2018 cash spend based on the re-normalised cash spend shares. We then multiplied these spend figures with the relevant carbon multipliers (**Table SI5**) to calculate carbon emissions for each of the 20 ATM spend categories. We then combined this dataset with TCS-level emission estimates, and aggregated these by individual and broad COICOP emission groups.

**References**

UK Finance (2020) ‘UK Consumer Payments 2020’. Available at: https://www.ukfinance.org.uk/policy-and-guidance/reports-publications/uk-consumer-payments-2020.
