# Supplementary material for: Estimating carbon footprints from large scale financial transaction data
Source: J Ind Ecol. 2022 Dec 27;27(1):56–70. doi: 10.1111/jiec.13351 (PMC13090182; doi:10.1111/jiec.13351)
Supplement: Supplementary file 3 — Supporting Information S6: This supporting information lists the 12 broad COICOP categories (Table SI2). [file 44498_2023_2701005_MOESM3_ESM.docx]

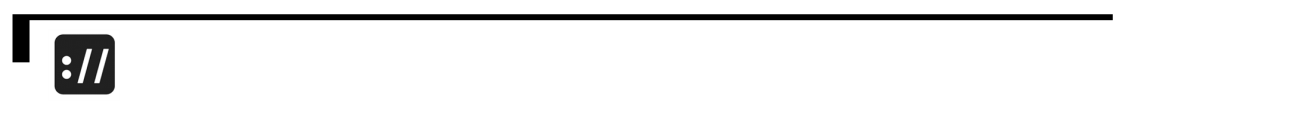


SUPPORTING INFORMATION FOR:

Trendl, A., Owen, A., Vomfell, L., Kilian, L., Gathergood, J., Stewart, N. & Leake, D. (2022.) Estimating carbon footprints from large scale financial transaction data. *Journal of Industrial Ecology.*


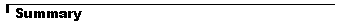


This supporting information provides an overview of the Kolmogorov-Smirnov statistics.


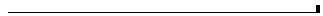


### Kolmogorov-Smirnov statistics

As part of validating the carbon footprint estimates calculated from spending data, we compared the distributions of the LBG and LCFS footprint estimates for different emission groups and at various levels of key demographic variables, including age, income, and region. We were primarily interested in measuring the similarity between LBG and LCFS footprint distributions.

We quantified this correspondence with the Kolmogorov-Smirnov statistic ($KS$), commonly used to compare distributions induced by different samples (Massey, 1951). In short, the $KS$ statistic captures the difference between two samples based on their respective empirical cumulative distribution functions (ECDFs).

Formally, given a collection of ordered data points $X_{1},X_{2},\ldots,X_{n}$, the ECDF $F_{n} \left( x \right)$ is defined as follows:

$$F_{n} \left( x \right) = \frac{Number of data points \leq x}{n}$$

An ECDF is a step function that increases by $\frac{1}{n}$ at the value of each ordered observed data point.

As illustrated by **Figure SI_3_KS_1**, assuming two ECDFs that correspond to two different samples (say, Sample 1 and Sample 2), visually, the Kolmogorov-Smirnov $KS$ statistic represents the largest absolute vertical distance between these ECDFs (see the right panel on **Figure SI_3_KS_1**). More formally, let $F_{1,n}$ give the ECDF of Sample 1 with $n$ observations and let $F_{2,m}$ give the ECDF of Sample 2 with $m$ observations and consider the union of the two samples resulting in $n+m = N$ data points, then the $KS$ statistic is defined as:

$$KS=\max_{1\leq i\leq N} | F_{1,n}\left( X_{i} \right)-F_{2,m}\left( X_{i} \right)|$$

The $KS$ statistic is bounded between 0 and 1, where 0 means that the two ECDFs (and therefore the underlying probability distributions) overlap perfectly, while a $KS$ statistic of 1 means that there is no overlap between the distributions.

*Figure SI_3_KS_1: Kolmogorov-Smirnov statistics*


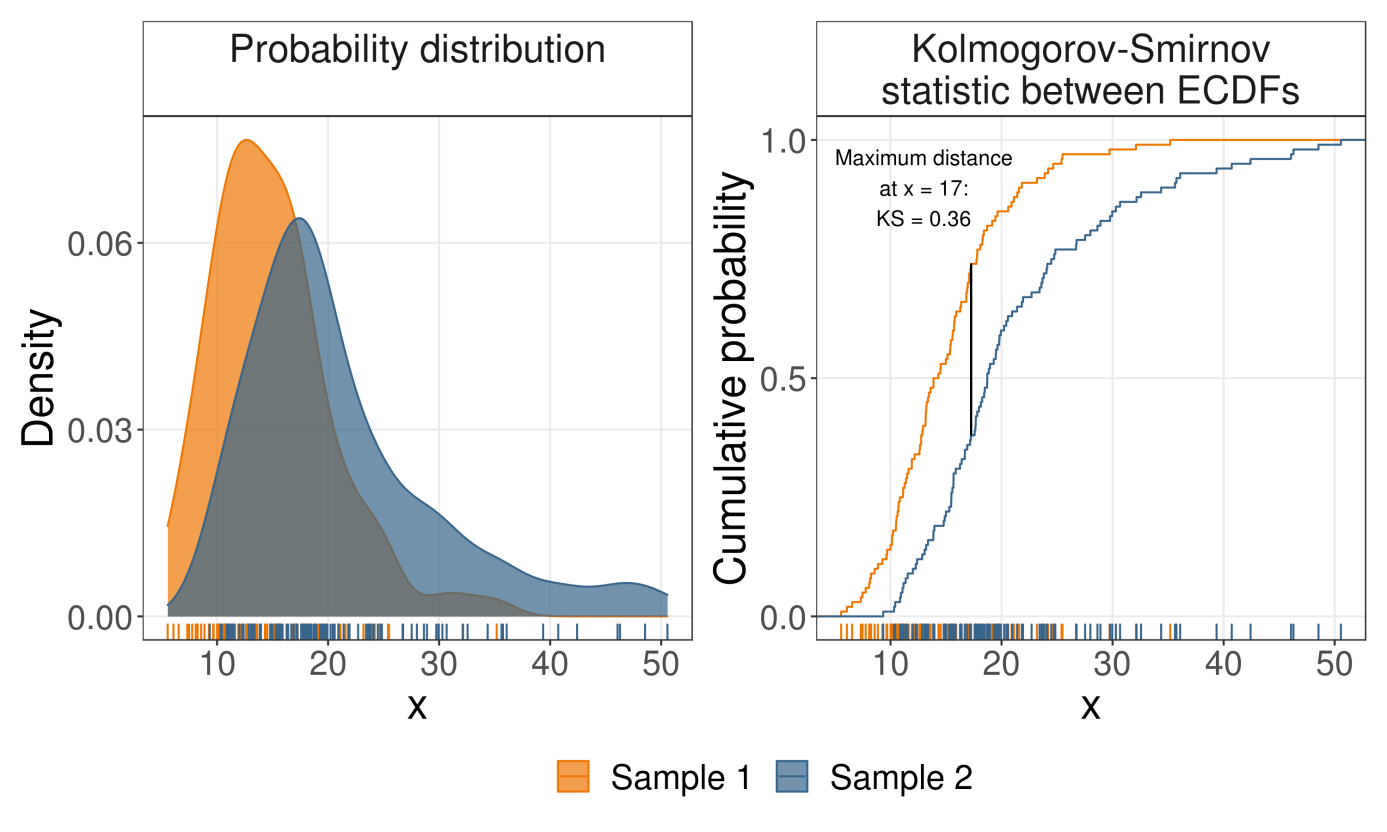


**References**

Massey, F. J. (1951) ‘The Kolmogorov-Smirnov Test for Goodness of Fit’, *Journal of the American Statistical Association*, 46(253). doi: 10.1080/01621459.1951.10500769.
