# Supplementary material for: Estimating carbon footprints from large scale financial transaction data
Source: J Ind Ecol. 2022 Dec 27;27(1):56–70. doi: 10.1111/jiec.13351 (PMC13090182; doi:10.1111/jiec.13351)
Supplement: Supplementary file 4 — Supporting Information S7: This supporting information lists the 5 TCS categories with the highest carbon multipliers (Table SI3). [file 44498_2023_2701005_MOESM4_ESM.docx]

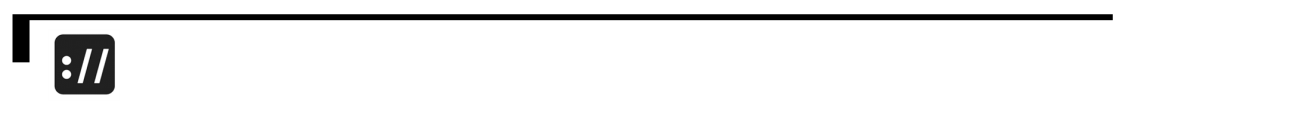


SUPPORTING INFORMATION FOR:

Trendl, A., Owen, A., Vomfell, L., Kilian, L., Gathergood, J., Stewart, N. & Leake, D. (2022.) Estimating carbon footprints from large scale financial transaction data. *Journal of Industrial Ecology.*


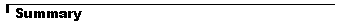


This supporting information provides an overview of the steps involved in our methodology.


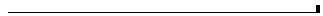


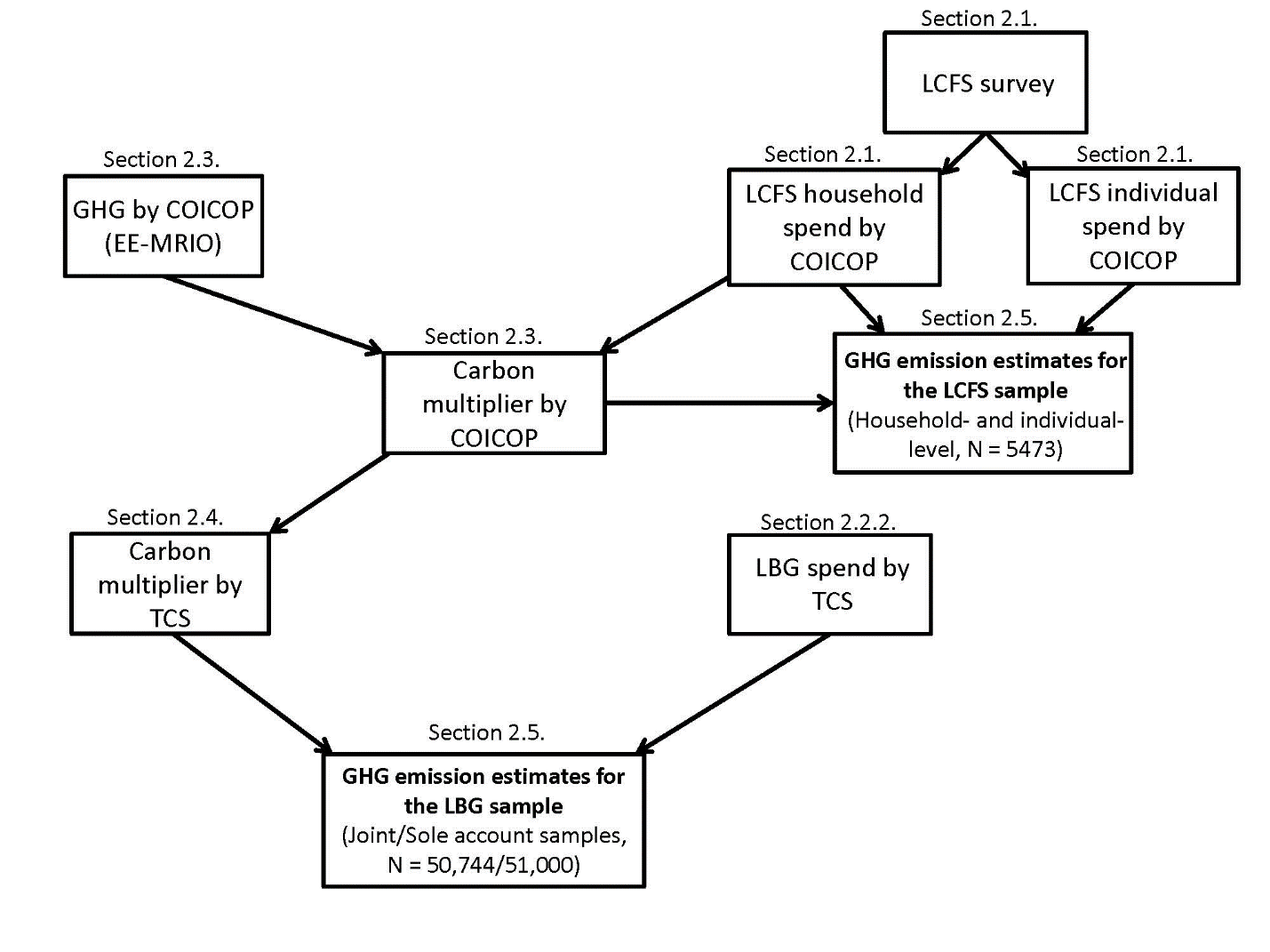


**Figure SI1.** An overview of the steps involved in our methodology.
